# A fully-mapped and energy-efficient FPGA accelerator for dual-function AI-based analysis of ECG

**DOI:** 10.3389/fphys.2023.1079503

**Published:** 2023-02-06

**Authors:** Wenhan Liu, Qianxi Guo, Siyun Chen, Sheng Chang, Hao Wang, Jin He, Qijun Huang

**Affiliations:** School of Physics and Technology, Wuhan University, Wuhan, China

**Keywords:** electrocardiagram (ECG), convolutional neural network (CNN), field programmable gate array (FPGA), signal processing, artificial intelligence (AI)

## Abstract

In this paper, a fully-mapped field programmable gate array (FPGA) accelerator is proposed for artificial intelligence (AI)-based analysis of electrocardiogram (ECG). It consists of a fully-mapped 1-D convolutional neural network (CNN) and a fully-mapped heart rate estimator, which constitute a complementary dual-function analysis. The fully-mapped design projects each layer of the 1-D CNN to a hardware module on an Intel Cyclone V FPGA, and a virtual flatten layer is proposed to effectively bridge the feature extraction layers and fully-connected layer. Also, the fully-mapped design maximizes computational parallelism to accelerate CNN inference. For the fully-mapped heart rate estimator, it performs pipelined transformations, self-adaptive threshold calculation, and heartbeat count on the FPGA, without multiplexed usage of hardware resources. Furthermore, heart rate calculation is elaborately analyzed and optimized to remove division and acceleration, resulting in an efficient method suitable for hardware implementation. According to our experiments on 1-D CNN, the accelerator can achieve 43.08× and 8.38× speedup compared with the software implementations on ARM-Cortex A53 quad-core processor and Intel Core i7-8700 CPU, respectively. For the heart rate estimator, the hardware implementations are 25.48× and 1.55× faster than the software implementations on the two aforementioned platforms. Surprisingly, the accelerator achieves an energy efficiency of 63.48 GOPS/W, which obviously surpasses existing studies. Considering its power consumption is only 67.74 mW, it may be more suitable for resource-limited applications, such as wearable and portable devices for ECG monitoring.

## 1 Introduction

Cardiovascular disease (CVD) has been one of the most critical threats to human health, as more than 30% of global deaths are proven relevant to it (WHO, 2021). Electrocardiogram (ECG) is an efficient diagnostic tool for CVD, which has been widely used and investigated by various medical institutions for decades. Nowadays, the development of the healthcare industry results in an increasing demand for continuous CVD monitoring outside hospitals, such as home-based or community-based monitoring (Faruk et al., 2021). In these scenarios, wearable or portable devices are employed to acquire ECGs. To alleviate the burden of cardiologists, intelligent algorithms are expected to further analyze these ECGs and alert users if necessary. The algorithms can be deployed on local devices (i.e., wearable or portable devices) and remote servers. In particular, remote deployment requires ECG data transmission *via* the Internet, which may cause delays in response and privacy violations. Thus, local deployment of algorithms has attracted much attention and exploration, since it makes on-device analysis possible and provides privacy protection (Raza et al., 2022). In addition, local analysis is not influenced by Internet access. This means that it can provide a more stable service for users.

There have been considerable studies on intelligent algorithms for ECG analysis. Conventional algorithms always concentrate on time-domain characteristics. The core challenge for conventional algorithms may be the precise detection and calculation of fiducial points or segments in ECG waveforms, such as R-peak detection, ST-segment detection, and QRS-interval calculation (Merdjanovska and Rashkovska, 2022). Heart rate calculation is one of the most common applications based on conventional algorithms. It usually performs R-peak detection at first. Then heart rate can be calculated based on RR-intervals. Several robust R-peak detection algorithms have been widely employed in heart rate calculation, such as Pan-Tompkins algorithm (Pan and Tompkins, 1985), Hamilton algorithm (Hamilton, 2002), Kalidas algorithm (Kalidas and Tamil, 2017), Neurokit algorithm (Makowski et al., 2021), etc. With the help of heart rate calculation, wearable or portable devices can alert users if the heart rate is out of the normal range. Recently, artificial intelligence (AI) has shown an impressive development. Its representative technique, deep learning (DL), has been applied in many fields, including ECG analysis (Liu et al., 2021). Specifically, multiple layer perceptron (MLP), convolutional neural network (CNN), and recurrent neural network (RNN) are widely adopted to diagnose CVDs using ECG, and achieve promising performances in detection of various CVDs (Liu et al., 2021). In general, these DL models have typical complex hierarchical architecture and require high-performance hardware to perform. Considering wearable and portable devices are usually developed using low-power edge devices with limited computational resources (Seneviratne et al., 2017), rapid on-device inference of DL models is a crucial challenge for a comprehensive CVD monitoring based on ECGs.

To accelerate ECG analysis on edge devices, a fully-mapped field programmable gate array (FPGA) accelerator for dual-function ECG analysis is proposed. The algorithms of ECG analysis include CVD diagnosis based on a lightweight 1-D CNN and heart rate estimating based on a self-adaptive R-peak detection. This is why the analysis is termed as a dual-function one. The motivation to develop this dual-function system is that the two algorithms are complementary as mentioned above. Implementing these two algorithms on FPGA provides a more comprehensive analysis and acceleration. In detail, the main contributions and advantages of this study are as follows:

(1) A fully-mapped implementation of 1-D CNN on FPGA is proposed to accelerate CVD diagnosis using ECGs. Unlike the existing studies, this study maps the full architecture of a 1-D CNN to FPGA rather than developing multiplexed computing units. Moreover, the virtual flatten layer is designed to effectively bridge the feature extraction stage (convolutional and pooling layers) and classification stage (fully-connected layers) of the CNN. Overall, this implementation achieves impressive acceleration and energy efficiency in our experiments.
(2) A self-adaptive heart rate estimator is developed on FPGA to accelerate heart rate calculation and complement the 1-D CNN in function. It consists of pipelined transformations, self-adaptive threshold, and heart rate calculation. Note that the calculation of the heart rate requires an accumulation of RR-intervals and a division between the number of sampling points in a minute and the average RR-interval. But the proposed method can remove the accumulation and division in hardware. It is also a fully-mapped hardware design as no unit is performed in a multiplexed manner. All these effective approaches make the heart rate estimator demonstrate high performance in deviation and acceleration.
(3) The implementations have good generalizations and compatibility. Both of the algorithms are evaluated on an ECG database containing more than 10,000 patients and they show good performances in CVD diagnosis and heart rate calculation. Furthermore, all the implementations are developed using Verilog HDL rather than high-level synthesis (HLS) tools relying on specific manufacturers (e.g., Xilinx, Intel, Lattice, etc.). This makes our implementations have good compatibility with devices from different manufacturers.

The rest of this paper is organized as follows. Section 2 introduces studies related to this work. Section 3 describes database, algorithms, and details about FPGA. In Section 4, evaluations on algorithm generalization and FPGA acceleration are explained. A brief discussion is performed in Section 5. Finally, Section 6 concludes the whole paper.

## 2 Related works

FPGA may be an appropriate choice to realize high-speed ECG analysis. It provides various configurable hardware units like logic elements, on-chip random access memory [RAM, which can also be configurated as read-only memory (ROM)], high-speed input/output pins, registers, and digital signal processing (DSP) block. By programming these hardware units, algorithms can be implemented in a parallel manner and performed in a pipeline manner. This allows FPGA to effectively accelerate algorithms. Although graphic processing units (GPU) also provide parallel acceleration for algorithms, FPGA is proved to be much more energy efficient (Ma et al., 2018), which can satisfy the power constraint of edge devices. In (Panigrahy et al., 2015), Panigraphy et al. employed Xilinx Virtex-5 FPGA to develop a heart rate monitoring system. They designed a new R-peak algorithm and evaluated its performance on the MIT-BIH database. Similarly, Agrawal et al. (Agrawal and Gawali, 2017) also implemented a peak detection algorithm on Xilinx Virtex-5 FPGA. Their approach can detect not only R peaks but also P and T peaks. To facilitate heart rate variability (HRV) analysis, Abdullah et al. (Abdullah and Abd, 2016) proposed a simple FPGA-based system to detect RR-intervals of ECGs. Nevertheless, these studies just used FPGA to perform their algorithms, no specific advantage of FPGA (high speed, high flexibility, etc.) over other platforms was revealed. In (Chen et al., 2020), Chen et al. designed a high-performance R-peak detection algorithm. They implemented it on Intel Cyclone-V FPGA and evaluated acceleration performance, which is a more comprehensive study than the aforementioned ones. However, their FPGA implementation only accelerated the algorithm by 13% compared with software implementation using Matlab. A more efficient FPGA-based accelerator for ECG analysis was proposed by Gu et al. (2016). Compared with software implementation using Matlab, it achieved a 6.69× speedup on feature extraction including R-peak detection and other fiducial points detection. But quantitative performance evaluations for the algorithms were not carried out (Gu et al., 2016).

Apart from the aforementioned applications, FPGA can also be employed to deploy and accelerate AI-based algorithms (especially various neural networks like MLP and CNN) for ECG analysis. Wess et al. (2017) used principal component analysis (PCA) and 3-layer MLP to detect arrhythmia. To deploy the MLP on a Xilinx Zynq FPGA, they optimized the non-linear activation functions by piecewise linear approximation. This method can reduce the amount of required resources by more than 80%. Similarly, Zairi et al. (2020) and Wang et al. (2017) also employed 3-layer MLPs to classify arrhythmia based on ECG beats, but they used discrete wavelet transform (DWT) for feature extraction. Moreover, the DWT and the MLP were jointly deployed on FPGAs to implement complete monitoring systems. Although impressive accuracies of arrhythmia detection were reported in these three representative MLP-based studies, only small-scale datasets were used to evaluate and optimize the algorithms. This may be difficult to reveal the actual feasibility of their systems. Furthermore, shallow neural network like 3-layer MLP has an inferior generalization to deep neural network in theory (LeCun et al., 2015). For deeper models, Wei et al. (2021) designed a 15-layer 1-D CNN to classify ECG beats into five classes, and a similar model is also explored (Lu et al., 2021). In detail, they developed generic convolutional and pooling layer processing units, and performed these units in a multiplexed way to complete CNN inference. This manner may be inspired by the FPGA accelerator design for CNN in computer vision (Ma et al., 2018). Although it can reduce resource consumption, it is not flexible enough to fully utilize the specific superiorities of FPGA (Gong et al., 2018). In addition, communications between computational units and on-chip/off-chip memories are too frequent in this manner, which may result in considerable power consumption (Ma et al., 2018). Overall, the aforementioned works focused on either ECG classification using neural networks or heart rate estimating based on R-peak detection. Although a neural network can detect CVDs for an ECG, it cannot directly estimate actual heart rate. On the other hand, heart rate can be estimated using an R-peak detection algorithm, but this algorithm cannot model the complex relationships between ECGs and corresponding CVDs. Implementing only one aspect on FPGA may be insufficient for real-world healthcare. The main purpose of this study is to address these limitations. It aims to implement a heart rate estimator and CNN-based ECG classifier in a fully-mapped manner. The CNN and heart rate estimator are intentionally designed to have their own hardware modules. There is no resource sharing between the CNN and the heart rate estimator. Furthermore, each unit of CNN or heart rate estimator has its own hardware module. Its advantages can result in impressive acceleration and power efficiency, which are presented in Section 4.

## 3 Materials and methods

### 3.1 Chapman ECG database

In this study, a large-scale ECG database (Zheng et al., 2020) proposed by Chapman University and Shaoxing People’s Hospital is used to develop and evaluate our algorithms. It contains 10,646 patients’ 12-lead records lasting 10 s, and the sampling rate is 500 Hz. Cardiologists have categorized the database into 11 rhythm classes, while a merged 4-class classification is recommended by (Zheng et al., 2020) to evaluate the generalization of a classification algorithm. These four classes include atrial fibrillation (AFIB), general supraventricular tachycardia (GSVT), sinus bradycardia (SB), and sinus rhythm (SR). Totally, the database has 2,225 AFIB patients, 2,307 GSVT patients, 3,888 SB patients, and 2,225 SR patients. Notably, a record is found to last only 3.85 s so it is removed here. As result, 10,645 records are used in this study.

Data preprocessing consists of 2 phases. First, all the records are downsampled from 500 Hz to 250 Hz. This can reduce computational burden and 250 Hz is enough to reveal ECG characteristics (Merdjanovska and Rashkovska, 2022). Second, each record is normalized by z-score. Given an ECG record *x*, normalized record **z** can be obtained by:

$$\begin{array}{c} z=\frac{x-\mu }{\delta } \end{array}$$ (1)

where *μ* and *δ* are the average value and standard deviation of ***x***, respectively. In this study, lead II is used to facilitate algorithm development, as it is the most widely used lead in ECG analysis (Merdjanovska and Rashkovska, 2022). Moreover, the database provides the actual heart rate for each record. This can be used for the evaluation of the heart rate estimator.

### 3.2 Algorithms

#### 3.2.1 1-D CNN

**FIGURE 1:**
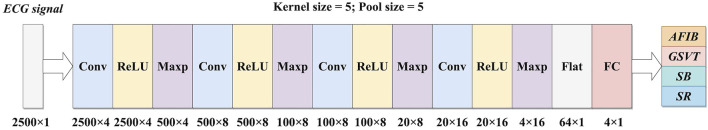
An overview of the proposed 1-D CNN. *Conv*: Convolutional layer; *Maxp*: Max-pooling layer; *Flat*: Flatten layer; *FC*: Fully-connected layer; *AFIB*: atrial fibrillation; *GSVT*: general supraventricular tachycardia; *SB*: sinus bradycardia; *SR*: sinus rhythm.

An overview of the proposed 1-D CNN architecture is shown in Figure 1. This CNN consists of four convolutional layers, four max-pooling layers, one flatten layer, and one fully-connected layer. The input of the CNN is a 10-s ECG record which contains 2,500 samples, while the output is a 4-element vector corresponding to the four target classes.

In particular, 1-D convolutional layer accounts for the most computation of the CNN. Let ***I***_***c***_ be the input, ***ω***_***c***_ be the kernel, ***b***_***c***_ be the bias of a 1-D convolutional layer, respectively, the output ***O***_***c***_ is obtained by:

$$\begin{array}{c} O_{c}\left[l\right]\left[n\right]=f\left(\overset{N_{c}-1}{\underset{n=0}{\sum }}\overset{M_{c}-1}{\underset{m=0}{\sum }}\overset{K_{c}-1}{\underset{k=0}{\sum }}I_{c}\left[l+k\right]\left[m\right]\times \omega_{c}\left[k\right]\left[n\right]+b_{c}\left[n\right]\right) \end{array}$$ (2)

where *K*_*c*_ is the length of the kernel. *M*_*c*_ and *N*_*c*_ are the numbers of input and output feature maps, respectively. Activation function *f* (˙) denotes rectified linear unit (ReLU) here. By training on large-scale datasets, convolutional layers are capable of extracting critical features related to specific CVDs, while max-pooling layers are employed to reduce feature dimension and achieve translation invariance. Actually, the max-pooling operation is to find the maximum value in a sliding window. It can be formulated as:

$$O_{p}\left[l\right]\left[n\right]=\max_{r=0,1,\ldots ,P_{w}-1}I_{p}\left[l\times P_{w}+r\right]\left[n\right]$$ (3)

where ***I***_***p***_ and ***O***_***p***_ are the input and output feature maps of the max-pooling layer, respectively. *P*_*w*_ denotes pooling size, i.e., the size of the sliding window. Convolutional and max-pooling layers are alternatively stacked to accomplish the feature extraction stage of the CNN. Then all feature maps are flattened to a feature vector ***V*** by the flatten layer. The fully-connected layer projects the feature vector to the final 4-element (corresponding to the four target classes) vector ***Y*** by:

$$\begin{array}{c} Y\left[n\right]=softmax\left(\overset{M_{v}}{\underset{m=0}{\sum }}V\left[m\right]\times \omega_{f}\left[m\right]\left[n\right]+b_{f}\left[n\right]\right) \end{array}$$ (4)

where *M*_*v*_ denotes the length of ***V***, ***ω***_***f***_ and ***b***_***f***_ are the weight and bias of the fully-connected layer, respectively. Here activation function *softmax* (˙) refers to the softmax function, which can generate a probability distribution for the target classes. The class with the maximum probability is regarded as the predicted class for the input ECG. As softmax is a monotonically increasing function, the class with the maximum probability must have the maximum value after the multiplications and accumulations in (4). Removing softmax does not influence the final predicted class. Additionally, exponent arithmetic is used in softmax, it is not suitable for hardware implementation. Thus, softmax is not implemented on FPGA in practice.

#### 3.2.2 Heart rate estimator

**FIGURE 2:**
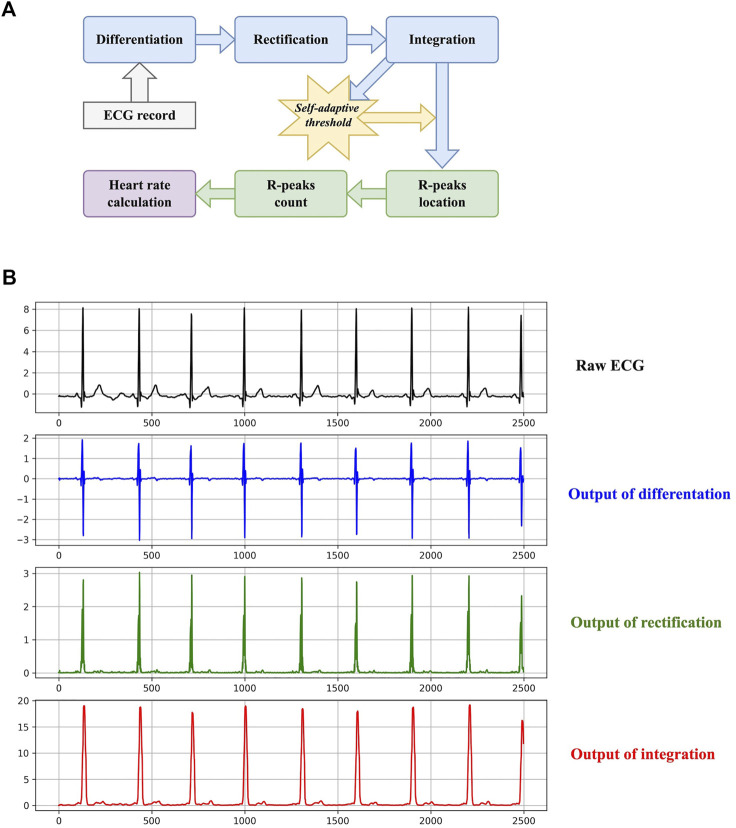
The method of the proposed heart rate estimator. **(A)** The diagram. **(B)** An example of the transformations presented in the diagram.

The basic workflow of heart rate calculation is to count R-peaks, calculate the mean RR-interval in a specific time range (10 s in this study), and then estimate the possible number of R-peaks in a minute. Although heart rate calculation is simple in methodology, it has been widely used in wearable and portable devices nowadays (Jeong et al., 2019). It provides a quantitative insight for heart activity frequency, which the 1-D CNN cannot. The diagram of the proposed method is shown in Figure 2.

The purpose of the first three steps is to transform an ECG to a signal with simpler waveforms, which is suitable for R-peak detection. Differentiation, rectification, and integration can be formulated as:

$$\begin{array}{c} D\left(t\right)=\frac{dx\left(t\right)}{dt}=x\left(t\right)-x\left(t-1\right) \end{array}$$ (5)

$$\begin{array}{c} R\left(t\right)=\left|D\left(t\right)\right| \end{array}$$ (6)

$$\begin{array}{c} S\left(t\right)=\overset{W}{\underset{w=0}{\sum }}R\left(t+w\right) \end{array}$$ (7)

where ***x*** denotes the input ECG record, ***D***, ***R***, and ***S*** denote the output of differentiation, rectification, and integration, respectively. *W* is the size of the sliding window when performing integration. It is set to 16 here. Figure 2 also plots an example of the three transformations. Regardless of negligible tiny waves, the final output signal ***S*** only contains sharp peaks corresponding to R peaks. Furthermore, the self-adaptive threshold *THR* is generated according to ***S***:

$$\begin{array}{c} THR=\gamma ∙\max_{t=0,1,\ldots ,M_{s}}S\left(t\right) \end{array}$$ (8)

where *M*_*s*_ is the length of ***S***, and *γ* is a hyperparameter to control the sensitivity of the threshold. It is set to 0.375 by trial and error. If the amplitude of a peak in ***S*** exceeds *THR*, it will be deemed to indicate an R peak in the raw ECG. It is worth noting that *THR* is not a fixed threshold and it varies according to the processing ECG. This is why it is termed a self-adaptive threshold. Additionally, the refractory period is introduced to reduce false detection. If the distance between two adjacent R-peak candidates is less than 0.24 s, only one candidate will be retained.

After R peaks are located, RR intervals can be calculated. A RR interval denotes the distance between two adjacent R peaks. Heart rate (beats per minute, *BPM*) is calculated using the average RR interval of the record:

$$\begin{array}{c} BPM=\frac{60∙f_{s}}{\frac{1}{N-1}\overset{N-1}{\underset{i=1}{\sum }}{RR}_{i}}=\frac{60∙f_{s}∙\left(N-1\right)}{\overset{N-1}{\underset{i=1}{\sum }}{RR}_{i}} \end{array}$$ (9)

where *N* refers to the number of R peaks in the record. Therefore, there are (*N*-1) RR intervals, and RR_*i*_ denotes the *i-*th RR interval. The sampling rate is represented by *f*_*s*_. It is equal to 250 Hz in this study.

### 3.3 Hardware design

#### 3.3.1 Model quantization

Usually, quantization can cause performance degradation compared with the model using float parameters, due to the information loss. A higher number of quantization bits can produce less performance degradation, as it can maintain more information of the original model (float). Meanwhile, a higher number of quantization bits requires more hardware resources to store parameters and perform calculations, as the number of bits for each parameter representation increases. Similarly, a lower number of quantization bits results in more performance degradation and less hardware resource cost. In particular, 8-bit quantization is the most commonly used method to quantize a neural network (Nagel et al., 2021). This means that each float value is represented by an 8-bit integer. Thus, 8-bit quantization is employed in this study.

For 1-D CNN, the parameters of each trainable layer (convolutional layer or fully-connected layer) are quantized by layer-wise symmetrical quantization. The model is trained using float weights in advance. Then the quantization is performed to make the weights and the input data become 8-bit integers. Once the input data and weights become integers, the feature maps derived from the calculations between input data and weights become integers, too. For the activation, the ReLU function is used. It only contains a comparison operation (> 0 or not). This can be directly applied to integers, and no additional quantization is performed for it. Given a ***ω*** containing the float parameters of a trainable layer, it is quantized by:

$$\begin{array}{c} \bar{\omega }=\left\lceil \frac{\omega }{{\left|\omega \right|}_{\max}}∙\left(2^{7}-1\right)+\frac{1}{2}\right\rceil \end{array}$$ (10)

where | ***ω*** |_*max*_ is the maximum absolute value in ***ω***. Moreover, float ECG data are quantized in a similar way. However, a convolutional layer can produce results exceeding the range of 8-bit representation by multiplications and accumulations. To guarantee that the input of the next convolutional layer can be represented by 8-bit integers, a rescaling is performed on the output of each convolutional layer:

$$\begin{array}{c} O^{\prime }\left[l\right]\left[n\right]=\frac{O\left[l\right]\left[n\right]}{2^{nb}}=\left(O\left[l\right]\left[n\right]\to nb\right),\\ nb=\left\lceil \log_{2}\frac{\max_{X}\left|O\right|}{2^{7}-1}\right\rceil \end{array}$$ (11)

$$\begin{array}{c} \bar{O}\left[l\right]\left[n\right]=\left\{\begin{array}{c} -\left(2^{7}-1\right),O^{\prime }\left[l\right]\left[n\right]<-\left(2^{7}-1\right)\\ O^{\prime }\left[l\right]\left[n\right],-\left(2^{7}-1\right)\leq O^{\prime }\left[l\right]\left[n\right]\leq \left(2^{7}-1\right)\\ \left(2^{7}-1\right),O^{\prime }\left[l\right]\left[n\right]>\left(2^{7}-1\right) \end{array}\right. \end{array}$$ (12)

where ***O*** ∈ℝ^*L*×*N*^ is the raw integer output of a convolutional layer. Function max_***X***_| ***O*** | is to find the maximum value of ***O*** across the whole dataset ***X***. Note that, ***O*** [*l*][*n*] divided by 2^*nb*^ is equivalent to performing *nb*-bit arithmetic right shift (→ *nb*) on ***O*** [*l*][*n*], so it is suitable for hardware implementation. Eq. 12 further truncates the output to ensure that it is in the target range.

For the heart rate estimator, only the calculation of the self-adaptive threshold [refer to (8)] requires quantization, as it uses float hyperparameter *γ* = 0.375. This can be replaced by arithmetic right shift and addition:

$$\begin{array}{c} THR=0.375∙S_{\max}=\left(\frac{1}{2^{2}}+\frac{1}{2^{3}}\right)S_{\max}\\ =\frac{S_{\max}}{2^{2}}+\frac{S_{\max}}{2^{3}}=\left(S_{\max}\to 2\right)+\left(S_{\max}\to 3\right) \end{array}$$ (13)

where ***S***_*max*_ is the maximum value of ***S***. Due to this conversion, multiplication is replaced by simpler operations (i.e., arithmetic right shift and addition), which can result in a more efficient hardware implementation. Indeed, quantization of the algorithms may lead to performance degradation. This will be explored in the next section.

#### 3.3.2 Fully-mapped 1-D CNN

As mentioned above, a fully-mapped design means that each layer/operation has its own hardware module on FPGA. Its advantages lie in two aspects. First, it allows to maximize the parallelism of operations, as mutual dependencies caused by multiplexed usage are removed. This can benefit the accelerating performance. Second, it reduces communications between computational units and memories, as parameter re-configuration required in multiplexed usage is eliminated. This can reduce the power consumption of the design (Gong et al., 2018; Ma et al., 2018). Detailed implementations are described in the following.

**FIGURE 3:**
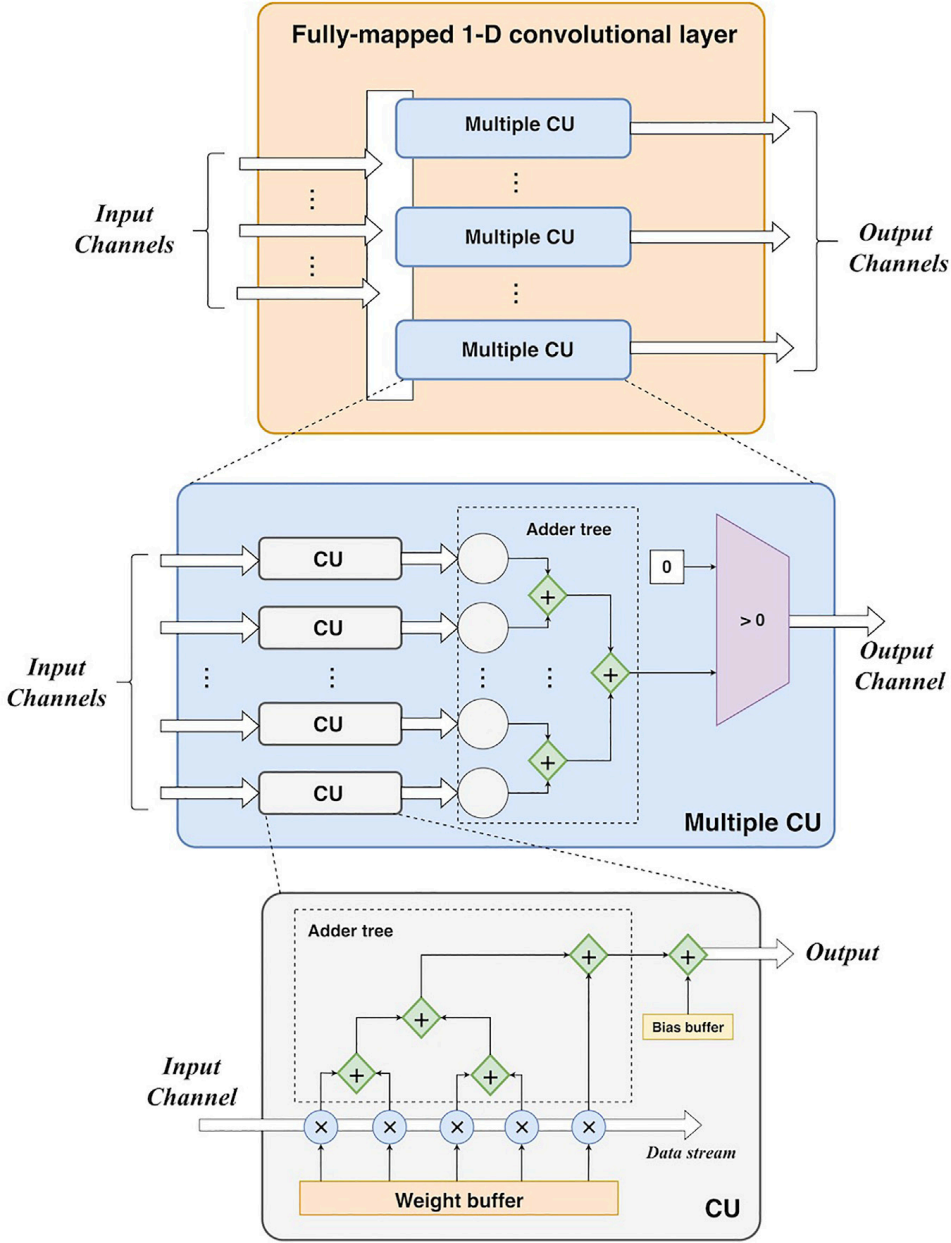
The hardware design of a fully-mapped 1-D convolutional layer. CU: convolutional unit.

1) *Fully-mapped 1-D convolutional layer*. Each channel of the input feature map has an independent input port. As a data stream, it is fed into its own convolutional unit including parallel multipliers and adder trees. As the kernel size of each convolutional layer is 5, there are five multipliers in a convolutional unit. For each multiplier, it consists of a combinational logic circuit of multiplication and a D-trigger for the result buffer for a better timing performance. For each clock cycle, it acquires two values to perform multiplication of them and stores the result in the D-trigger. Then it takes an additional clock cycle to output this result. There is a queue register for data buffering before the multiplication. This queue register stores five values from the input stream. For each clock cycle, the register loads a new value and removes the oldest value. Each value in the register can be directly obtained using its index. Also, the weights are stored in the registers that can be directly extracted by using an index (like an array data structure). Thus, the data access of the input data and weights does not cause additional latencies. Then the multiplications are conducted by five multipliers in a parallel manner. For accumulation, a 3-level adder tree is developed. The adders at the same level can work in parallel, and the adders at different levels work in a pipelined manner. However, if the adder tree is treated as a whole, it works serially as the input data is fed into it as a stream. The number of the output port is determined by the number of output channels. ReLUs can be easily implemented by comparers (an input value vs. 0). In summary, this fully-mapped design parallelizes a convolutional layer at the channel level. Furthermore, it unrolls each convolutional operation using parallel multipliers and adder trees. The high parallelism can enable high-performance acceleration in theory. Figure 3 shows the design of the convolutional layer.

To generate the whole output feature map, the convolutional layer must process the whole input feature map. Since the operations can cause latencies, resulting in more clock cycles to yield the whole output. In general, the number of clock cycles of the *i-*th convolutional layer can be calculated as:

$$\begin{array}{c} {Cycle}_{c}^{i}=L_{c}^{i}+1_{mul}+\left\lceil \log_{2}K_{c}\right\rceil +\left\lceil \log_{2}N_{c}^{i}\right\rceil +P_{c}^{i}+1_{relu}+1_{rescale} \end{array}$$ (14)

where *Li c* is the length of the input feature map, *K*_*c*_ is the length of the kernel, and *Ni c* is the number of output feature maps. 1_*mul*_ and 1_*relu*_ are the 1-cycle latencies of the multipliers and ReLUs, respectively. Since a rescaling should be performed to maintain the 8-bit output, it leads to 1-cycle latency (1_*rescale*_). The third element denotes the latency of the adder tree in a convolutional unit (CU) as shown in Figure 3, whereas the fourth element refers to the latency of the adder tree in the summation of multiple CU. *Pi c* denotes the padding size before the convolution, resulting in additional *Pi c* clock cycles.

**FIGURE 4:**
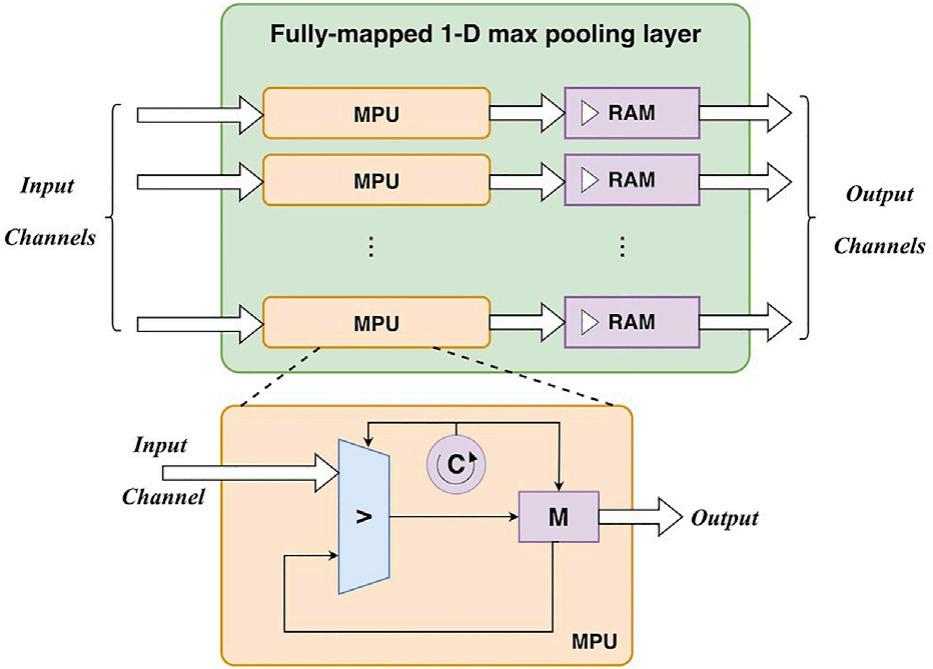
The hardware design of a fully-mapped 1-D max-pooling layer. MPU: max-pooling unit.

2) *Fully-mapped 1-D max-pooling layer*. A convolutional layer (including activation) and a max-pooling layer can constitute a basic unit of the 1-D CNN. The output of the convolutional layer can be directly fed into the max-pooling layer for subsampling. Similarly, a max-pooling layer is also parallelized at the channel level. There is a max-pooling unit for each input/output channel. Particularly, the max-pooling operation requires to find the maximum value in a specific window containing *pool_size* samples. As input data are fed into the unit as a stream, a loop counter (0 ∼ *pool_size*) is employed to locate the pooling windows. With the help of a register for maximum value storage, pooling results can be obtained after traversing all samples in the window. It is worth noting that the data rate is divided by *pool_size* after pooling, as *pool_size* samples only generate one output sample. To maintain the same data rate for the following layers, the output of a max-pooling layer is buffered to block RAMs at first. Then the output is read from these RAMs at the same data rate for the following process. The design of the max-pooling layer is depicted in Figure 4.

Based on the input feature map, the max-pooling operation generate an output value for every *P*_*w*_ input values, i.e., *P*_*w*_ cycles. Thus, it causes a *P*_*w*_ -cycle latency. In addition, the comparison operation in the max-pooling also results in a 1-cycle latency (1_*comp*_). Thus, the number of clock cycles of the *i*-th max-pooling layer can be calculated as:

$$\begin{array}{c} {Cycle}_{p}^{i}=L_{p}^{i}+P_{w}+1_{comp} \end{array}$$ (15)

where *Li p* is the length of the input feature map. Note that there is no buffer between a convolutional layer and a max-pooling layer. The output of the convolutional layer is fed into the max-pooling layer. Thus, the total cycles of a unit including a convolutional layer and a max-pooling layer are:

$$\begin{array}{c} {Cycle}_{u}^{i}={Cycle}_{c}^{i}+P_{w}+1_{comp} \end{array}$$ (16)

3) *Virtual flatten layer*. Unlike other layers, flatten layer does not have any parameters. It only reorganizes feature maps to a feature vector. For hardware implementation, this reorganization requires considerable data buffer and communications in theory, which can cause additional latency and power consumption. However, the flattening operation can be eliminated by calculating the corresponding position in the feature vector for each sample of the feature maps in advance. Let ***I***_***t***_∈ℝ^*L*×*N*^ denote the input feature maps of the flatten layer (i.e., the output feature maps of the last max-pooling layer with *N* channels), the corresponding position of ***I***_***t***_ [*l*][*n*] in the feature vector can be calculated as:

$$\begin{array}{c} Pos\left(I_{t}\left[l\right]\left[n\right]\right)=l\times N+n \end{array}$$ (17)

**FIGURE 5:**
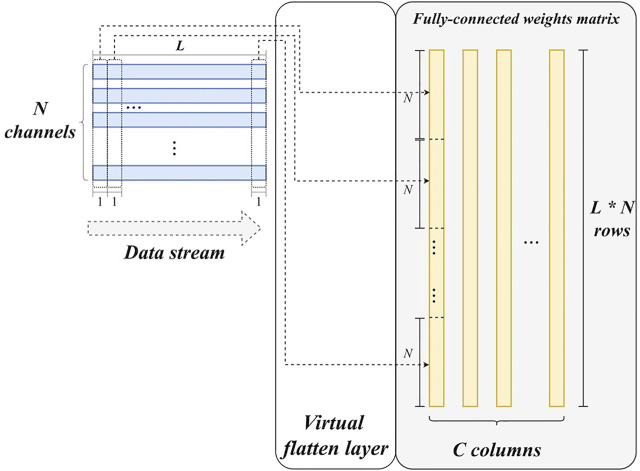
The hardware design of the virtual flatten layer.

Figure 5 also illustrates this operation. Based on this, feature maps can immediately match the weight matrix of the following fully-connected layer and then enable the matrix multiplication. To summarize, there is no corresponding hardware module for flatten layer, although flatten operation has been performed in an implicit way. This is why it is termed a virtual flatten layer. Obviously, this design can benefit the resource utilization and power consumption of our system.

**FIGURE 6:**
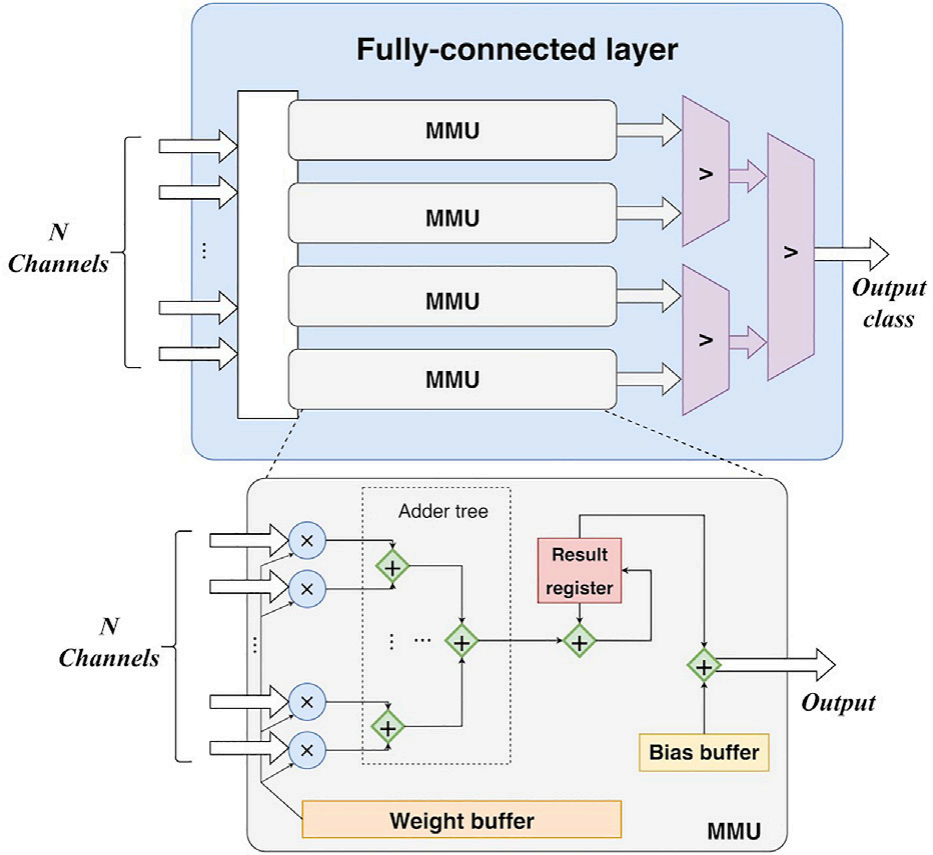
The hardware design of the fully-connected layer. MMU: matrix multiplication unit. *N*-channel input is fed into each MMU in the fully-connected layer. In other words, all MMUs share the same *N*-channel input and perform calculations in parallel.

4) *Fully-connected layer*. The main operation in the fully-connected layer is matrix multiplication. All weights are buffered in register arrays. The parallelism of the matrix multiplication is determined by the number of input feature map channels. In detail, feature maps are synchronously read from *N* block RAMs, corresponding to the *N* output channels of the final max-pooling layer. Thus, there are *N* values fed into the fully-connected layer for each clock cycle. Accordingly, *N* multipliers and (*N*-1) adders are employed to accomplish multiplications and accumulations in a parallel manner, as shown in Figure 6. In this study, *N* is set to 16. Furthermore, the calculation for each column of the weight matrix is independent, making parallelization along columns possible. A predicted class can be obtained by three comparers after the matrix multiplication.

Concretely, the fully-connected layer reads data from the RAMs of the last max-pooling layer. According to Figures 5, 6, given an *L* as the length of the output feature map of the last max-pooling layer, the cycles of the fully-connected layer can be formulated as:

$$\begin{array}{c} {Cycle}_{f}=L+1_{mul}+\left\lceil \log_{2}N\right\rceil +1_{csum}+2_{comp} \end{array}$$ (18)

where 1_*mul*_ and 1_*csum*_ are the 1-cycle latencies caused by multiplications and cumulative summation in the matrix multiplication. 2_*comp*_ denotes the 2 1-cycle latencies from the three comparers after the matrix multiplication as shown in Figure 6. The third element denotes the latency from the adder tree. The total cycles of the 1-D CNN can be calculated as:

$$\begin{array}{c} {Cycle}_{CNN}=\sum_{i}{Cycle}_{u}^{i}+{Cycle}_{f} \end{array}$$ (19)

Notably, the fully-mapped implementation of 1-D CNN is a generalized method for the design of hardware accelerators. In brief, it aims to map each unit of the 1-D CNN to a hardware module without multiplex usage, maximizing the parallelism of the calculation. Thus, it does not rely on the specific CNN architecture presented in the manuscript. To adjust the proposed design to a new 1-D CNN model, the number of input/output channels and the number of computing units (convolutional unit in Figure 3, max-pooling unit in Figure 4, matrix multiplication unit in Figure 6) can be modified to match its architecture.

#### 3.3.3 Fully-mapped self-adaptive heart rate estimator

**FIGURE 7:**
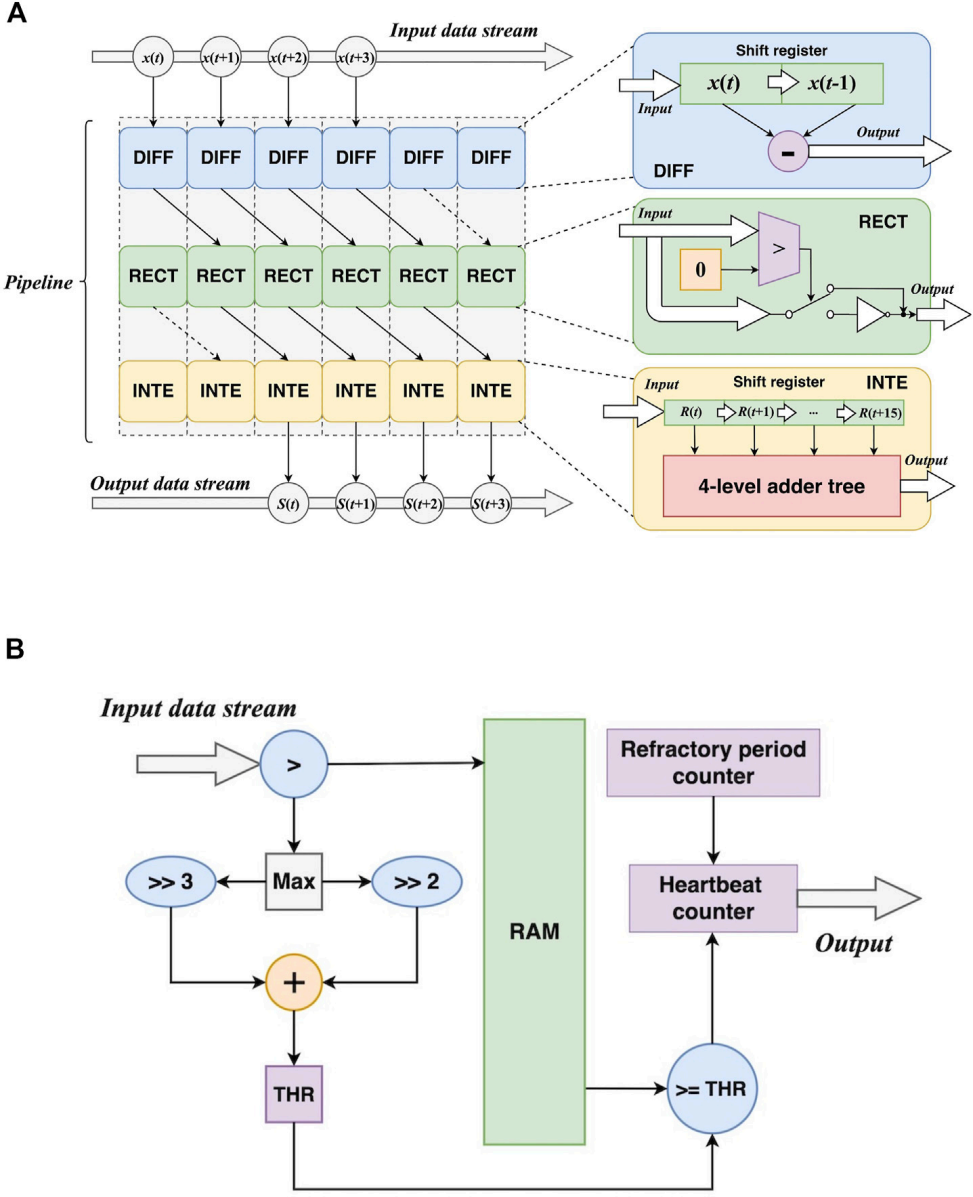
Hardware design of the preparation before heart rate calculation. **(A)** The pipelined design of the transformations DIFF: differentiation; RECT: rectification; INTE: integration. **(B)** The design of self-adaptive threshold calculation.

1) *Pipelined transformations*. For differentiation, it can be implemented by a 2-element shift register and a subtracter. Rectification needs a comparer and an inverter to detect negative values and invert them. Integration employs a 16-element shift register and a 4-level adder tree to implement accumulation. Furthermore, the three transformations can be performed in a pipelined manner. Figure 7A shows the architectures of the three transformations and the pipeline. In this way, the transformations can be synchronously performed, resulting in a speedup. Let *L* be the length of the signal, it takes *L* cycles to feed the signal into the transformations. Also, the operations in the three transformations result in latencies. The total cycles can be formulated as:

$$\begin{array}{c} {Cycle}_{tran}=L+1_{diff}+1_{rect}+\left\lceil \log_{2}W\right\rceil \end{array}$$ (20)

where 1_*diff*_ and 1_*rect*_ are the 1-cycle latencies from differentiation and rectification, respectively. The integration can take ⌈log_2_ *W*⌉ cycles to implement the accumulation *via* the adder tree, and *W* is the width of the window.

2) *Self-adaptive threshold.* According to (8), the calculation of the self-adaptive threshold depends on the maximum value of the transformed signal. This requires a traversal of the signal. In addition, all values of the signal should be buffered for the following steps. After obtaining the maximum value, the threshold can be calculated by arithmetic right shift and addition. Then it is used as a criterion for R-peak localization, and the number of R peaks is provided by a counter. In addition, a loop counter is employed to measure the distance between two adjacent R peaks and apply the refractory period mechanism. Figure 7B also depicts the aforementioned modules.

For the self-adaptive threshold, the operations consist of comparison, arithmetic right shift, and addition. The arithmetic right shift and the addition constitute a logic to calculate the threshold based on the maximum value from the comparison. These leads to 1-cycle latencies (1_*comp*_ and 1_*thr*_):

$$\begin{array}{c} {Cycle}_{thr}=L_{t}+1_{comp}+1_{thr} \end{array}$$ (21)

where *L*_*t*_ is the length of the transformed signal. As the transformed signal is directly fed into the calculation of the self-adaptive threshold. Thus, the preparation process before the beat count can be formulated as:

$$\begin{array}{c} {Cycle}_{prep}={Cycle}_{tran}+1_{comp}+1_{thr} \end{array}$$ (22)

When applying the threshold, an additional comparison is required to highlight the part exceeding the threshold with a 1-cycle latency (1_*highlight*_). The loop counter for refractory period calculation and the heartbeat counter can introduce extra 1-cycle latencies (1_*ref*_ and 1_*beat*_). The cycles can be obtained as:

$$\begin{array}{c} {Cycle}_{beat}=L_{t}+1_{highlight}+1_{ref}+1_{beat} \end{array}$$ (23)

3) *Heart rate calculation without accumulation and division.* Considering (9), the main challenges for hardware implementation of heart rate calculation can be summarized as two aspects, including the accumulation of RR intervals and the division. Direct implementations of these operations may cause considerable hardware resource consumption and additional latency (Lu et al., 2021). Nevertheless, more efficient implementations are proposed in this study to overcome the challenges by eliminating accumulation and division.

Given a 10-s ECG record containing *N* R peaks, locations of these R peaks are represented by *P*_1_∼*P*_*N*_, so the *i*th RR interval is RR_*i*_ = *P*_*i*+1_-*P*_*i*_. Accumulation of the RR intervals can be formulated as:

$$\begin{array}{l} \overset{N-1}{\underset{i=1}{\sum }}RR_{i}=\overset{N-1}{\underset{i=1}{\sum }}\left(P_{i+1}-P_{i}\right)\\ =P_{N}-P_{N-1}+P_{N-1}+\ldots -P_{2}+P_{2}-P_{1}\\ =P_{N}-P_{1} \end{array}$$ (24)

Therefore, only locations of the first and the last R peaks are required to calculate the RR interval accumulation. Based on (15), (9) can be rewritten as:

$$\begin{array}{c} BPM=\frac{60∙f_{s}∙\left(N-1\right)}{P_{N}-P_{1}}=\frac{60∙f_{s}}{P_{N}-P_{1}}∙\left(N-1\right)\\ =r\left(∆P_{N,1}\right)∙\left(N-1\right) \end{array}$$ (25)

$$\begin{array}{c} r\left(∆P_{N,1}\right)=\frac{60∙f_{s}}{P_{N}-P_{1}}=\frac{C_{s}}{∆P_{N,1}} \end{array}$$ (26)

where *C*_*s*_ is a constant. As *f*_*s*_ is equal to 250 Hz here, the value of *C*_*s*_ is 15,000 = 60 × 250. ∆*P*_*N*,1_ = *P*_*N*_-*P*_1_ represents the number of sampling points between the first R-peak position and the last one. It must be a natural number, and its range can be inferred as:

$$\begin{array}{c} 0.24∙f_{s}<∆P_{N,1}<t∙f_{s} \end{array}$$ (27)

where *t* = 10 denotes the length of an ECG record, and (0.24・*f*_*s*_) represents the refractory period. Thus, all possible values of ∆*P*_*N*,1_ are the 24,939 integers between 60 and 25,000. This makes *r* (∆*P*_*N*,1_) have finite results, which can be stored in a ROM in advance. As *r* (∆*P*_*N*,1_) is not always an integer, all possible values are represented as 16-bit fixed-point numbers. After that, the heart rate can be calculated without division. As formulated in (16), the calculation is accomplished by a ROM-read operation [*r* (∆*P*_*N*,1_)] and multiplication with (*N*-1).

As mentioned above, the heart rate calculation is transformed to a read ROM-read operation and a multiplication. As this multiplication is performed using fix-point numbers, a truncation operation is required to obtain the integer part of the result. These three operations can cause 1-cycle latencies. The cycles for heart rate calculation can be obtained as:

$$\begin{array}{c} {Cycle}_{bpm}=L_{t}+1_{rom}+1_{mul}+1_{trun} \end{array}$$ (28)

The heart rate calculation is directly performed on the output of the beat count module, thus the cycles of these two modules are:

$$\begin{array}{c} {Cycle}_{hrc}={Cycle}_{beat}+1_{rom}+1_{mul}+1_{trun} \end{array}$$ (29)

To summarize, challenges originating from accumulation and division are overcome by our effective conversions. This makes heart rate calculation more suitable for hardware implementation on FPGA. The total cycles of the heart rate estimator can be calculated as:

$$\begin{array}{c} {Cycle}_{HR}={Cycle}_{prep}+{Cycle}_{hrc} \end{array}$$ (30)

## 4 Results

### 4.1 1-D CNN training and test

To train the 1-D CNN, the Chapman database introduced in Section 2 is randomly divided into a training set (80% of the records) and a test set (20% of the records). In addition, 10% of the training records are used as a validation set for hyperparameter adjustment and model selection. Adam optimizer is employed during the training procedure. The learning rate and the batch size are set to 0.001 and 128, respectively. The model is trained for 50 epochs and a checkpoint is saved for each epoch. Cross entropy is employed to measure the loss during the training. The checkpoint with the lowest loss on the validation set is selected for performance evaluation on the test set. The model and the training procedure are implemented using Python and Keras with a TensorFlow backend (v2.5.0). It is worth noting that there is no patient overlap between training, validation, and test set. The model is tested on unseen patients. This inter-patient evaluation can make results more reliable (da et al., 2016).

Accuracy (Acc) and macro-average F1 score (F1-macro) are employed as performance metrics. For Acc, it is a ratio between the number of true classified records (*T*) and the number of false classified records (*F*) as:

$$\begin{array}{c} Acc=\frac{T}{T+F} \end{array}$$ (31)

To calculate F1-macro, F1 score for each class should be obtained. Given a class as positive class, other classes are gathered as a negative class, F1 score is a harmonious average of recall and precision:

$$\begin{array}{c} recall=\frac{TP}{TP+FN} \end{array}$$ (32)

$$\begin{array}{c} precision=\frac{TP}{TP+FP} \end{array}$$ (33)

$$\begin{array}{c} F1=\frac{2\times recall\times precision}{recall+precision} \end{array}$$ (34)

Where TP and FP denote true positive and false positive, respectively. FN denotes false negative. After that, F1-macro can be calculated as:

$$\begin{array}{c} F1-macro=\frac{\overset{N_{C}}{\underset{i=1}{\sum }}{F1}_{i}}{N_{C}} \end{array}$$ (35)

where *N*_*C*_ is the number of classes and F1_*i*_ denotes the F1 score of the *i*th class. Each experiment uses both Acc and F1-macro to measure the model generalization comprehensively.

**FIGURE 8:**
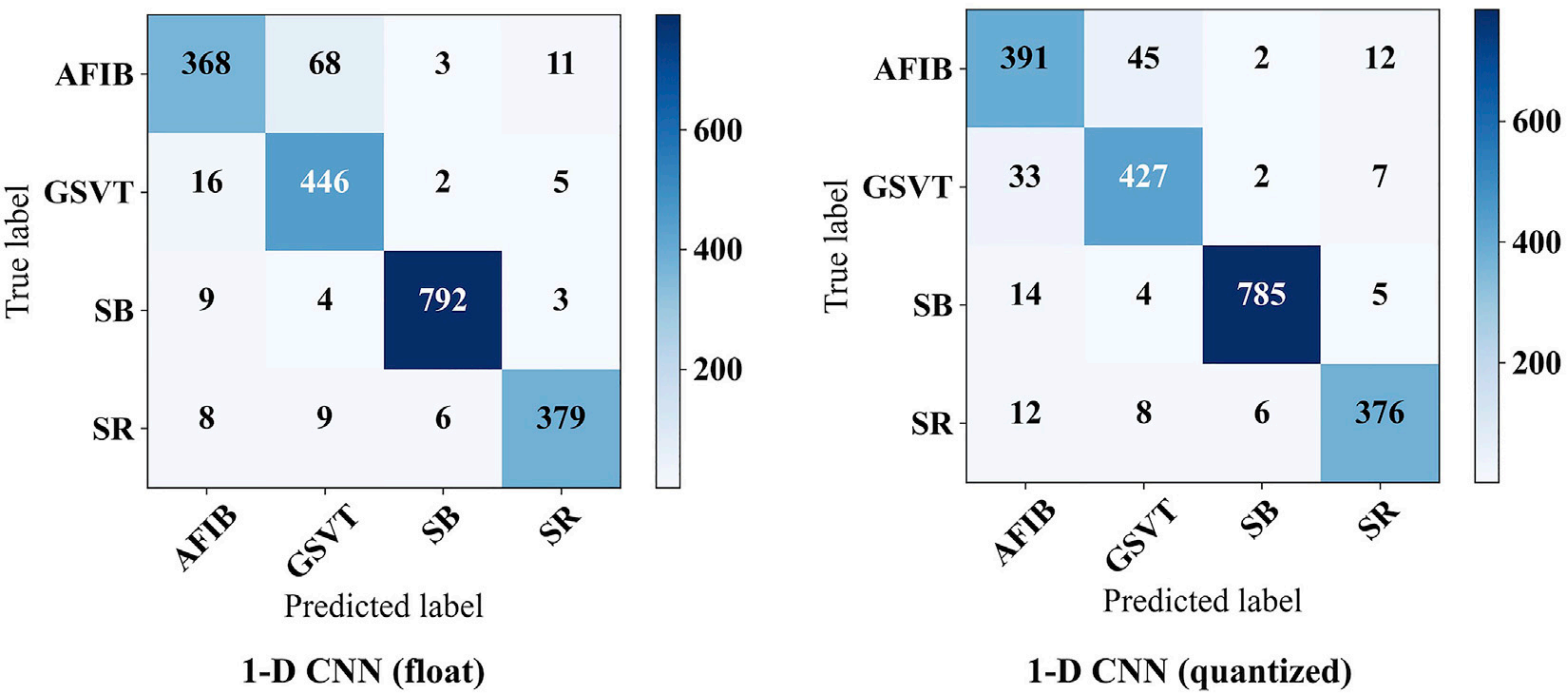
The confusion matrices on test set.

**FIGURE 9:**
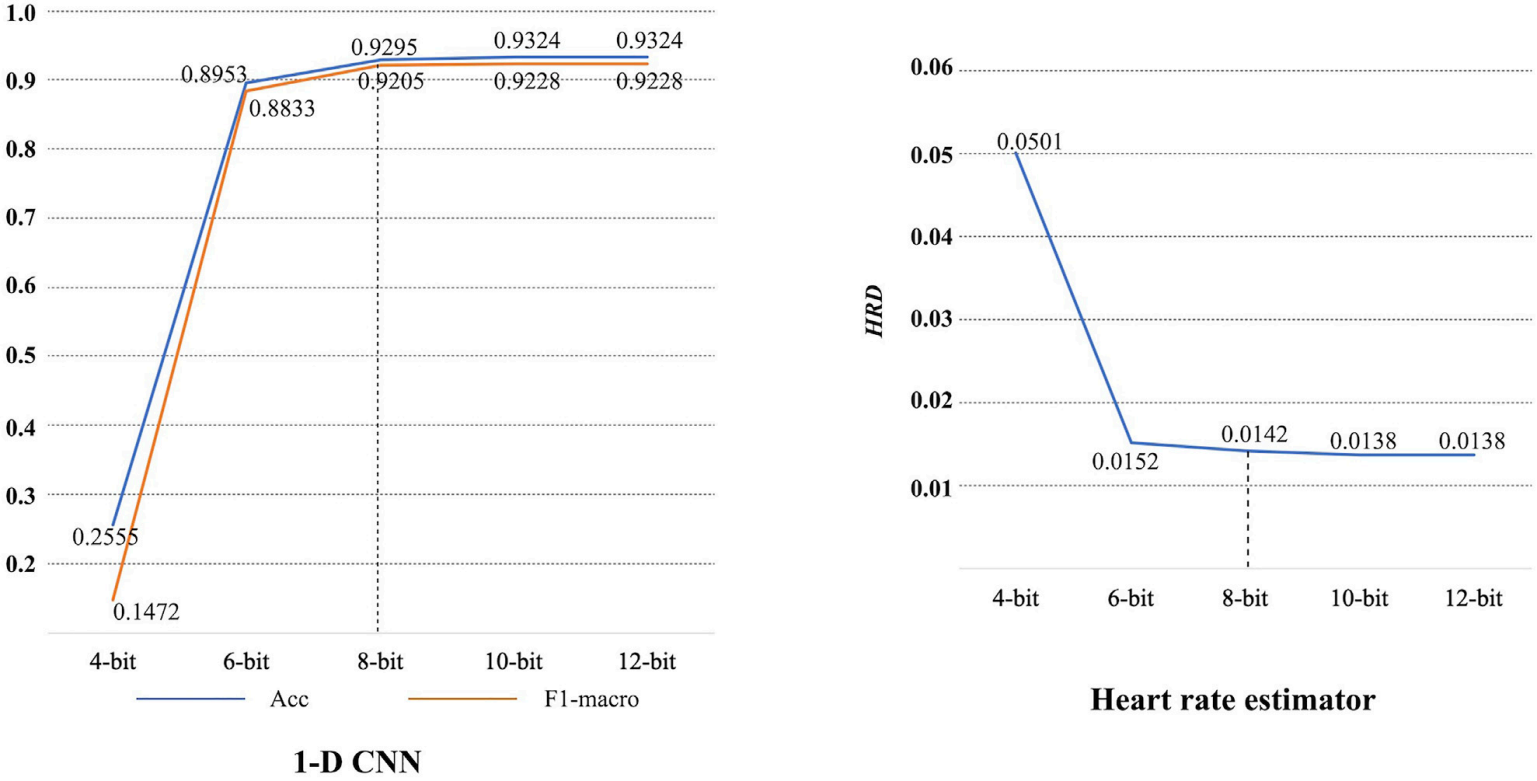
Performance degradation caused by quantization.

Figure 8 shows the confusion matrix on the test set. Based on this, it can be obtained that the 1-D CNN achieves an accuracy of 0.9324 and an F1-macro of 0.9228. Note that the parameters of trained 1-D CNN are stored as float numbers. As mentioned in Section 3.1, they should be quantized to 8-bit integer numbers for hardware implementation. Thus, the quantized 1-D CNN is also evaluated. The confusion matrix on the test set is also displayed in Figure 8. The Acc and F1-macro are 0.9295 and 0.9205, respectively. To further justify the 8-bit quantization, 4-bit, 6-bit, 10-bit, and 12-bit quantization methods are also carried out to explore their influence. The performance changes caused by these quantization methods are shown in Figure 9. It can be seen that the quantization employing less than 8 bits shows obvious performance degradation. Although the methods employing more than 8 bits can achieve the same performance as the algorithms based on float numbers, they can cause additional hardware resource costs. As shown in Figure 9, Acc and F1-macro only decrease by 0.0029 and 0.0023 based on 8-bit quantization, respectively. This demonstrates that the quantization method is effective.

### 4.2 Heart rate estimator test

The proposed heart rate estimator is implemented using C++. A heart rate deviation (*HRD*) is employed to measure the performance of the heart rate estimator. Let *h* be the actual heart rate of a record and‾*h* be the estimated heart rate, *HRD* can be obtained by:

$$\begin{array}{c} HRD=\left|\frac{h-\bar{h}}{h}\right| \end{array}$$ (36)

Lower *HRD* always means better performance. All records in the Chapman database are processed by the proposed heart rate estimator, then an average *HRD* is calculated. The proposed heart rate estimator achieves an average *HRD* of 0.0138. After 8-bit quantization, the average *HRD* increases to 0.0142. A performance degradation of 0.0004 can be obtained. It is also acceptable since the degradation is negligible. 4-bit, 6-bit, 10-bit, and 12-bit quantization methods are also investigated here, as shown in Figure 9. Similarly, 8-bit quantization can achieve a good balance between performance and hardware resource cost.

### 4.3 Hardware acceleration

**FIGURE 10:**
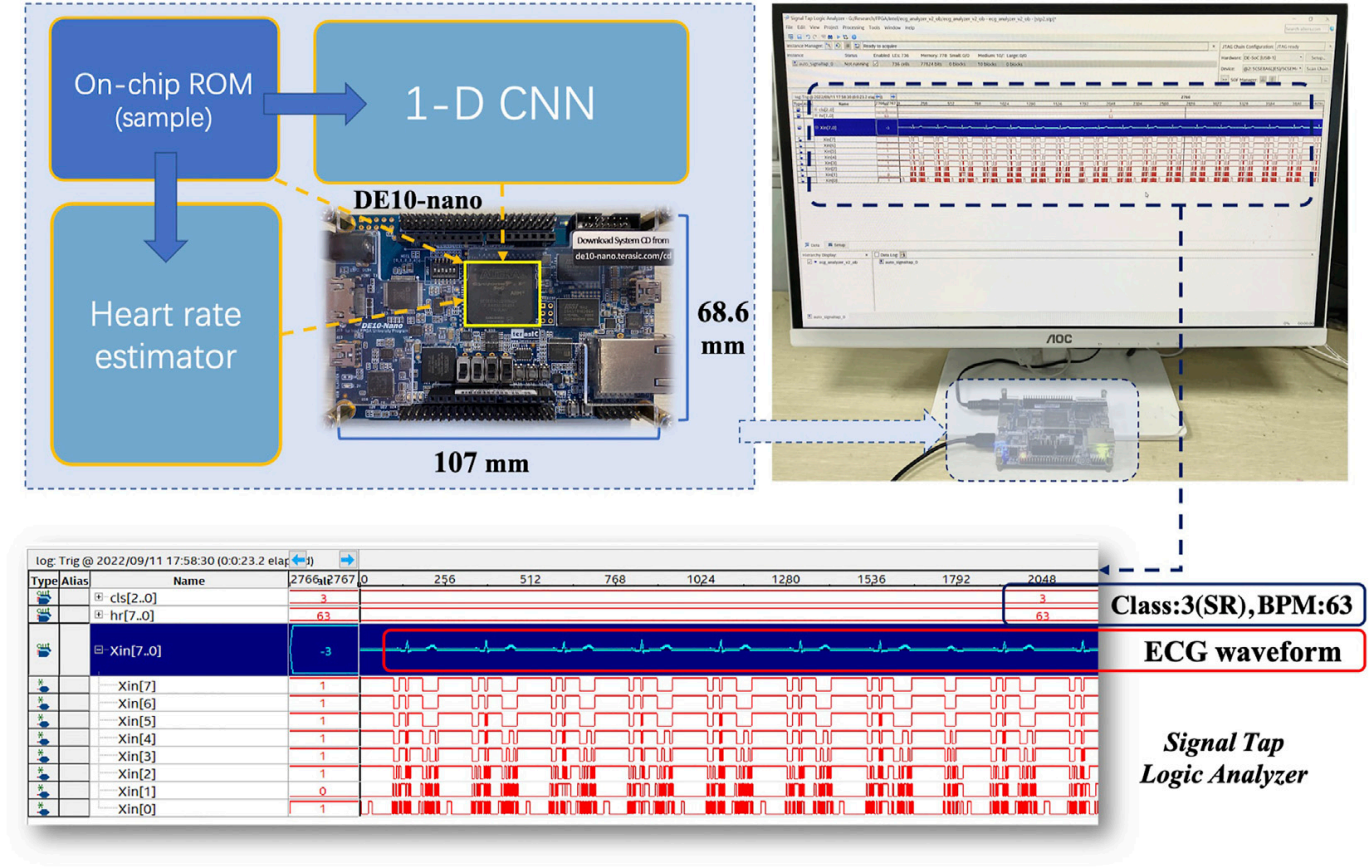
Test on DE10-nano development board.

Both 1-D CNN and heart rate estimator are implemented using an Intel Cyclone V FPGA (5CSEBA6U23I7) on the Terasic DE10-nano development board. The FPGA has 41,910 adaptive logic modules (ALMs), 83,820 registers, 5662720 bits on-chip memory, and 112 DSP block. Notably, Cyclone V FPGA is optimized for low-power and low-cost applications (Intel, 2018), which is suitable for continuous ECG monitoring and analysis. The hardware project is established and synthesized using Quartus Prime software (v18.1). All modules share a global clock of 50 MHz. As shown in Figure 10, an on-board test is conducted after programming the FPGA. ECG signals are stored in the on-chip memory. To access the acceleration performance, the signals are read from the memory and synchronously fed into the 1-D CNN and heart rate estimator. Signal Tap Logic Analyzer embedded in Quartus Prime can capture and display real-time signal behavior, helping debug hardware design. According to the analyzer, our design works as expected on the DE10-nano development board. There are two baseline platforms used for comparison, including a Windows PC with an Intel Core i7-8700 CPU and a Raspberry Pi 3B with an ARM Cortex-A53 quad-core processor. The software implementations of the algorithms are performed on the two baseline platforms. Then a latency comparison is carried out between baselines and our hardware design. Notably, only the quantized algorithms are deployed on the FPGA.

**FIGURE 11:**
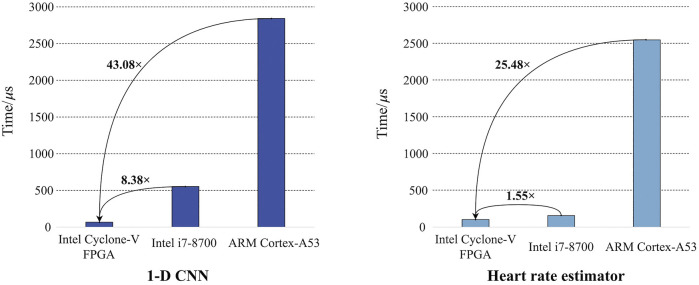
Acceleration performance for 1-D CNN and heart rate estimator.

For the FPGA implementation of 1-D CNN, it takes 66 *μ* *s* to process a 10-s ECG record according to our experiment. By contrast, the inference latencies of the software implementations (Keras) on Intel Core i7 8,700 and ARM Cortex-A53 are 553 *μ* *s* and 2,843 *µ*s, respectively. Keras is a high-performance software deep-learning framework. It can automatically use multiple CPU cores or threads for model inference to optimize the speed (Abadi et al., 2016). In addition, an advanced execution paradigm based on a dataflow graph is employed to facilitate co-current running (Abadi et al., 2016). Due to its efficient framework, Keras is widely used in algorithm research and deployment. Therefore, it is reasonable to compare the proposed hardware design with the software design based on Keras to validate the advantages of the proposed hardware design. The comparison is shown in Figure 11. The FPGA implementation achieves 43.08× speedup compared with the software implementation on ARM Cortex-A53. Considering the Intel Core i7-8,700 baseline, a more powerful CPU for PC, the FPGA implementation still achieves 8.38× speedup. Similarly, the software implementation of the heart rate estimator is performed and examined on the baseline platforms. It takes 155 *μ*s and 2,548 *μ*s to process an ECG record on Intel Core i7-8,700 and ARM Cortex-A53, respectively. However, the inference time of the FPGA implementation is only 100 *μ*s. Unlike CNN, the heart rate estimator does not have a specific framework for its software implementation. C/C++ may be the most efficient software language, except for low-level programming languages (Oualline, 2003), like Assembly language. Therefore, C++ is used to implement the heart rate estimator, and no additional optimization is used. In addition, C/C++ is widely used in embedded system development (Oualline, 2003), so it is also reasonable to compare its C/C++ implementation with the proposed hardware design. Comparison is also presented in Figure 11. The FPGA implementation is 1.55× and 25.48× faster than Intel Core-i7 8,700 and ARM Cortex-A53, respectively. As FPGA can perform integer calculation/operation without any information loss, the algorithm performances of the actual implementations on FPGA are consistent with those of the quantized algorithms shown in Figures 8, 9. To summarize, all results reveal the advantages of FPGA on parallel computation. Our hardware design can effectively utilize these advantages and achieve obvious speedup.

Power consumption is also estimated to verify power efficiency. Based on the Power Analyzer embedded in Quartus Prime, the FPGA implementation consumes 67.74 mW at a clock frequency of 50 MHz. It is much lower than the power consumption of Intel Core i7-8,700 (65 W) and ARM Cortex-A53 (3.7 W). This demonstrates that the FPGA implementation has better power efficiency than the baselines. The acceleration does not introduce considerable power consumption.

## 5 Discussion

To demonstrate the advantage of the proposed 1-D CNN, the neural networks for ECG classification in (Zairi et al., 2020), (Wess et al., 2017), and (Wei et al., 2021) are reproduced to perform the classification described in Section 2. Although there have been some complex models employed for the classification of the Chapman database (Yildirim et al., 2020; Baygin et al., 2021; Murat et al., 2021), they do not consider the efficient inference and FPGA deployment of the model. Thus, these models are not involved in the comparison. Performances of the existing models are listed in Table 1. Obviously, the proposed 1-D CNN outperforms the MLPs in (Wess et al., 2017) and (Zairi et al., 2020) by a considerable margin. Moreover, 1-D ECGs can be directly fed into the proposed 1-D CNN for classification. No extra preprocessing and feature extraction are required. Compared with the CNN in (Wei et al., 2021), our 1-D CNN achieves comparable results but has fewer parameters. Also, the proposed 1-D CNN is robust to the quantization operation, and only a slight performance degradation is obtained. These make our 1-D CNN more lightweight and suitable for implementation on resource-limited platforms. For the heart rate estimator, several commonly used R-peak detection algorithms are employed to calculate heart rate (Pan and Tompkins, 1985; Hamilton, 2002; Lourenço et al., 2012; Kalidas and Tamil, 2017; Makowski et al., 2021) and compare with the proposed one. Using the Chapman database, their performances are evaluated and listed in Table 2. The proposed heart rate estimator achieves the lowest HRD. This indicates its impressive adaptability to different patients.

**TABLE 1:** Classification performance comparison.

	Acc	F1-macro
Zairi et al. (2020)	0.4955	0.3911
Wess et al. (2017)	0.6195	0.5583
Wei et al. (2021)	0.9314	0.9242
Proposed (float)	**0.9324**	**0.9228**
Proposed (quantized)	**0.9295**	**0.9205**

Bold values refer to the performances of the proposed method.

**TABLE 2:** Heart rate estimation performance comparison.

	*HRD*
Pan and Tompkins, (1985)	0.0353
Hamilton, (2002)	0.0633
Lourenço et al. (2012)	0.0550
Kalidas and Tamil, (2017)	0.0223
Makowski et al. (2021)	0.0296
Proposed (float)	**0.0138**
Proposed (quantized)	**0.0142**

Bold values refer to the performances of the proposed method.

Energy efficiency measured by GOPS/W is always employed to compare different FPGA implementations. GOPS/W means Giga operations per second with a power consumption of 1 W. This metric alleviates the influence of model differences and makes the comparison fairer. Table 3 lists several representative studies on FPGA accelerators for ECG classification. Carreras et al. optimized the temporal convolutional network (TCN) inference on FPGA (Carreras et al., 2020). For ECG classification, their method can achieve an energy efficiency of 33.8 GOPS/W with a latency of 0.017 s. A similar performance is also obtained in (Wei et al., 2021). In (Srivastava et al., 2022), the power consumption of the probabilistic neural network (PNN) on FPGA is only 25 mW, which is much lower than the proposed method. But its latency is found to be 17 s when processing a 10-s ECG record, whereas the latency of the proposed method is only 66 *μ*s. For (Ran et al., 2022), FPGA was jointly employed with an ARM processor to classify ECGs. But the usage of ARM leads to an obvious increase in power consumption. In addition, it seems that the MLP used by (Wess et al., 2017) is too simple to fully utilize the advantages of FPGA. Low power efficiency is obtained in the experiments. However, the proposed accelerator achieves 87.42 GOPS/W and obviously outperforms the existing studies. The main reason is that the fully-mapped design can achieve higher parallelism. Also, it allows to configure all the parameters in advance, reducing memory communication during algorithm inference. This helps improve the power consumption of the design, as described in Section 3.

**TABLE 3:** Comparison with existing FPGA accelerators about ECG classification.

	Carreras et al. (2020)	Ran et al. (2022)	Srivastava et al. (2022)	Wess et al. (2017)	Wei et al. (2021)	Proposed
Platform	Zynq-7020	Zynq-7020	Artix-7	Zynq-7020	Zynq-7045	Cyclone V-5CSE
Model	TCN	CNN	PNN	MLP	1-D CNN	1-D CNN
Resource utilization	LUT:80.76%	LUT:47.1%	Reg:1.5%	LUT:3.6%	LUT:1.1%	ALM:51%
	Reg: 25.34%	Reg:19.7%	IOB:89%	Reg:1.8%	DSP:10.67%	Reg:86%
	Mem:91.4%	Mem:95%	BUFG:3%	DSP:14.5%		Mem:0.5%
	DSP: 100%	DSP:68.6%				DSP:39%
Clock (MHz)	120	100	100	100	200	50
Quantization	16-bit	8-bit	NA	24-bit	16-bit	8-bit
Latency s)	0.017	2.895	17	9.9 × 10^−7^	NA	6.6 × 10^−5^
Power W)	3.3	2.81	0.025	0.124	0.79	0.066
Operations (GOP)	NA	NA	NA	1.86 × 10^−7^	1.028 × 10^−3^	3.8 × 10^−4^
Efficiency (GOPS/W)	33.8	NA	NA	0.54	33.67	87.42

NA: Not Available; LUT: Look-up Table; Reg: Register; Mem: On-chip Memory; ALM: Adaptive Logic Module; IOB: Input-Output Block. BUFG: Buffer Global.

Also, a comparison about FPGA implementations of heart rate estimators is presented in Table 4. Except for the work proposed by Chen et al. (2020) each work successfully developed a low-cost heart rate estimator. Agrawal and Gawali (2017) achieved the lowest latency of all the listed studies, but this may be due to a high clock frequency of 287.505 MHz. Although they did not report the power of their design, a high clock frequency usually leads to a dramatic increase in power consumption. Abdullah et al. directly implemented a heart rate monitoring algorithm on a Spantan-3A FPGA, no extensive analysis about power/latency is presented. The methods proposed by Panigraphy et al. (2015) and Meddah et al. (2019) can perform real-time heart rate calculation, and lower power may be a special advantage of the hardware module designed in. However, they did not provide precise latencies of their systems. Considering the proposed one, it simultaneously achieves a low latency of 100 us and a low power of 1.88 mW. Unlike the work also based on Cyclone V FPGA (Chen et al., 2020), it costs fewer hardware resources. Therefore, the proposed hardware design shows obvious advantages over the existing works.

**TABLE 4:** Comparison with existing FPGA accelerators about heart rate estimation.

	Panigrahy et al. (2015)	Abdullah and Abd, (2016)	Agrawal and Gawali, (2017)	Meddah et al. (2019)	Chen et al. (2020)	Proposed
Platform	Virtex-5	Spantan-3A	Virtex-5	Artix-7	Cyclone V-5CSE	Cyclone V-5CSE
Resource utilization	LUT:29%	LUT: 1%	LUT:0.3%	LUT:10%	ALM:70%	ALM:0.57%
	Reg: 0.9%	Reg:1%	Reg:0.17%	Reg:1%	Mem:6%	Reg:0.79%
	IOB:16%	Mem:5%	Mem:1%	DSP:4%	DSP:100%	Mem:2.1%
	BUFG: 3%	IOB:10%	IOB:10%	IOB:11%		DSP:0.89%
		BUFG:12%	BUFG:3%			
Clock (MHz)	NA	NA	287.505	3.936	50	50
Quantization	16-bit	16-bit	8-bit	NA	NA	8-bit
Latency s)	NA	NA	4.7 × 10^−7^	NA	0.007	0.001
Power W)	NA	NA	NA	0.042	NA	1.88 × 10^−3^

NA: Not Available; LUT: Look-up Table; Reg: Register; Mem: On-chip Memory; ALM: Adaptive Logic Module; IOB: Input-Output Block. BUFG: Buffer Global.

In summary, the proposed design can simultaneously perform classification and heart rate estimation rather than only focus on classification/heart rate monitoring like other studies. If the 1-D CNN and heart rate estimator are simultaneously performed, the power consumption and the energy efficiency are 67.74 mW and 63.48 GOPS/W, respectively. Unlike the studies based on HLS (Wess et al., 2017; Ran et al., 2022), the proposed implementation is developed using Verilog HDL, corresponding to a pure RTL description. It does not rely on the HLS tool provided by a specific manufacturer, which means that it has advantages in flexibility and compatibility. Furthermore, it can be also wrapped as an Intellectual Property (IP) core with different interfaces and applied to various applications. Since the proposed implementation achieves lower power consumption than other studies, it may be more suitable for lightweight applications, such as wearable and portable devices for ECG monitoring.

## 6 Conclusion

This paper proposes a fully-mapped FPGA accelerator for ECG processing. It can not only classify ECG but also estimate heart rate. A 1-D CNN is proposed for the classification and implemented on FPGA in a fully-mapped manner. Each layer has its hardware module except for the virtual flatten layer. To complement the 1-D CNN, a fully-mapped heart rate estimator is employed to calculate heart rate using ECG. Each phase of the estimator corresponds to a specific hardware module on FPGA. According to the experiments, the proposed accelerator achieves significant speedup with low power consumption. This demonstrates that it has impressive energy efficiency, which outperforms existing studies. Considering the lower power consumption, the proposed accelerator can be applied to wearable and portable ECG monitoring devices. This can help the devices process ECG faster with limited resources. In the future, fully-mapped developments of more advanced neural networks and heart rate estimators will be explored. Furthermore, the accelerator will be integrated into a real-world ECG monitoring system for latency improvements. The code will be made available at https://github.com/Aiwiscal/ECG-AI-Accelerator.

## Data Availability

Publicly available datasets were analyzed in this study. This data can be found here: https://figshare.com/collections/ChapmanECG/4560497/2.
